# An optoacoustic imaging feature set to characterise blood vessels surrounding benign and malignant breast lesions

**DOI:** 10.1016/j.pacs.2022.100383

**Published:** 2022-07-04

**Authors:** O. Abeyakoon, R. Woitek, M.G. Wallis, P.L. Moyle, S. Morscher, N. Dahlhaus, S.J. Ford, N.C. Burton, R. Manavaki, I.A. Mendichovszky, J. Joseph, I. Quiros-Gonzalez, S.E. Bohndiek, F.J. Gilbert

**Affiliations:** a Department of Radiology, University of Cambridge, Cambridge Biomedical Campus, Hills Road, Cambridge CB2 0QQ, UK; b Cambridge Breast Unit, Cambridge University Hospitals NHS Foundation Trust, Cambridge CB2 0QQ, UK; c iThera Medical GmbH, Zielstattstrasse 13, Munich 81379, Germany; d Department of Nuclear Medicine, Cambridge University Hospitals Foundation Trust, Cambridge CB2 0QQ, UK; e Department of Physics, University of Cambridge, JJ Thomson Avenue, Cambridge CB3 0HE, UK; f Cancer Research UK Cambridge Institute, University of Cambridge, Robinson Way, Cambridge CB2 0RE, UK

**Keywords:** Breast cancer, Photoacoustic imaging, Hybrid imaging, Ultrasound

## Abstract

Combining optoacoustic (OA) imaging with ultrasound (US) enables visualisation of functional blood vasculature in breast lesions by OA to be overlaid with the morphological information of US. Here, we develop a simple OA feature set to differentiate benign and malignant breast lesions. 94 female patients with benign, indeterminate or suspicious lesions were recruited and underwent OA-US. An OA-US imaging feature set was developed using images from the first 38 patients, which contained 14 malignant and 8 benign solid lesions. Two independent radiologists blindly scored the OA-US images of a further 56 patients, which included 31 malignant and 13 benign solid lesions, with a sensitivity of 96.8% and specificity of 84.6%. Our findings indicate that OA-US can reveal vascular patterns of breast lesions that indicate malignancy using a simple feature set based on single wavelength OA data, which is therefore amenable to application in low resource settings for breast cancer management.

## 1 Introduction

Breast cancer is the most common malignant disease in women, with high morbidity, mortality, and socioeconomic burdens [1]. Breast cancer is a global disease with almost 50% of cases and 58% of deaths occurring in less developed countries [1]. Furthermore, low-income countries have survival rates below 40%, while high-income and middle-income countries achieve survival rates of 80% and 60%, respectively. These differences in survival rates can be partly explained by the discrepancies in early diagnosis (such as lack of screening programmes) and treatment facilities.

Clinical breast imaging methods include mammography, ultrasound (US) and magnetic resonance imaging (MRI). The sensitivity of mammography is dependent on breast density, with 90% achievable in a fatty breast compared to only 60% in the dense breast [2]. The sensitivity of US is not as high and sonographic features alone do not always allow differentiation between benign and malignant lesions, resulting in the need for biopsy or follow up of solid lesions as recommended in the BI-RADS lexicon [3]. A simple and low-cost detection method that could broadly facilitate an accurate diagnosis of breast cancer in more of the population, especially in countries without ready access to routine histopathology services, has the potential to increase early detection and improve survival from breast cancer.

The stimulation of neoangiogenesis is considered a rate-limiting step in breast cancer progression with prognostic significance [4]. Imaging features relating to neoangiogenesis have been widely explored as breast cancer biomarkers, which distinguish malignant and benign breast lesions. While standard-of-care mammography and US are used to visualise breast anatomy and identify morphological features, they are not sensitive to early functional changes associated with angiogenesis. Doppler techniques can be added to a normal B-mode US to provide information on vascularity [5], but are limited in breast cancer due to technical factors such as low flow rate [6]. Various MRI methods are available that detect and quantify angiogenesis [7], for example, blood oxygen level dependent and susceptibility weighted MRI, although these are specialist sequences that are not widely available. Although tumour vasculature can be highlighted using dynamic contrast enhanced methods, quantification of these data adds complexity to the clinical procedure and data interpretation, hence is not undertaken routinely in the breast [8], [9].

Optoacoustic (OA) imaging is currently being evaluated in clinical feasibility studies for breast cancer diagnosis and staging [10]. This potentially low cost technique [11] is based on the absorption of pulsed light irradiation by chromophores in the tissue of interest and the resulting generation of broadband acoustic pressure waves, which are detected using US transducers and converted into images. The range of wavelengths in the near-infrared window provides image contrast dominated by the presence of the chromophores oxy- and deoxy-haemoglobin. By imaging at specific wavelengths targeting the differential absorption spectra of oxy- and deoxy-haemoglobin, surrogate measures of haemoglobin content and oxygenation can be obtained [12].

A range of OA systems have been developed to exploit the biological differences in vasculature between benign and malignant lesions for diagnostic purposes. Handheld linear [13], [14] and curvilinear [15], [16] array systems have been built, as well as planar [17], [18] and ring/cup [19], [20], [21], [22] shaped systems, which are comprehensively reviewed in Manohar & Dantuma [10]. While initial clinical studies focused on technological developments with limited patient numbers, OA has been applied in larger breast imaging trials. For example, two multicentre studies based on a commercial instrument [23] considered the ability of OA to upgrade or downgrade BI-RADS 4a (suspicious) lesions [13], [14]. These studies used a 30-feature set derived from surrogate measures of oxy- and deoxy-haemoglobin to perform the lesion classification, however, the increase in sensitivity and specificity afforded by the 30 feature approach has not yet reached a level to obviate the need for biopsy. Attempts were also made to use the 30-feature set to differentiate between molecular subtypes of breast cancer [24], however, these are in their infancy and have not influenced the molecular classification of breast cancer diagnosed by tissue sampling.

Despite the promise of OA revealed by earlier studies, practical clinical application of the technology in a broader range of healthcare settings, including low-resource settings, could be facilitated by the simplification of the data acquisition and image interpretation, particularly when considering operator and reader training. Here, we sought to create a simple feature set using single wavelength OA data obtained from an integrated OA-US imaging system, which could be easily learnt and applied, with the goal of enabling differentiation of benign from malignant breast disease across a range of healthcare settings. Using the OA feature set, two readers were trained and then asked to independently and blindly score unseen lesions. Adding OA data to US showed a clear improvement in diagnostic specificity relative to US alone.

## 2 Materials and methods

### 2.1 Clinical study

This cross-sectional study was performed between March 2016 and July 2017 following approval by the East of England Cambridge South Research Committee (REF: 16/EE/0052) as a basic science study in human participants. Written informed consent was obtained from all study participants.

Female patients presenting with benign, indeterminate or suspicious abnormalities on clinical examination, mammography or US were recruited through the Breast Unit of the Cambridge University Hospitals NHS Foundation Trust. The exclusion criteria were pregnancy, lactation, vulnerable patient groups (e.g. inability to give consent), bruising, and skin disease/tattoos over the breast, which could interfere with the optoacoustic acquisition. OA-US imaging was performed during the clinic visit. Consecutive patients were recruited on days when there was scanner availability and workflow in the clinic allowed extended time to include an OA-US scan. All OA-US scans were performed by a radiologist with 5 years of experience in breast imaging and prior experience in OA-US imaging (Dr Oshaani Abeyakoon, first author) [25]. The radiologist performing the OA-US scans had no access to histopathology information at the time of imaging.

### 2.2 OA-US imaging

OA images were acquired at 800 nm, with the patient positioned supine on an US examination couch during the examination. The lesion previously identified by clinical examination or conventional mammography or ultrasound in the symptomatic breast clinic was assessed using a hybrid OA-US acquisition. Imaging took place before core biopsy or one week later, provided that there was no history of post-biopsy haematoma or visible skin discoloration. No patients examined had marker clips present. If more than one lesion was present, their locations were clearly identified and examined.

OA-US was performed with an MSOT EIP or MSOT Acuity Echo prototype (iThera Medical GmbH, Munich, Germany). The MSOT EIP [25], [26], or Experimental Imaging Platform, was the first generation version of the prototype OA-US device for clinical research; the MSOT Acuity Echo prototype was a second generation device that incorporated features required for certification as a medical device. From a data acquisition perspective, the internal components were similar between the devices. At the time of the study, neither device had received regulatory approval. The MSOT Acuity Echo has subsequently received CE certification. Both systems generated nanosecond excitation laser pulses using an OPO pumped by a Nd:YAG laser (Innolas GmbH) at a repetition rate of 20 Hz. Laser light was delivered via a custom-made fiber bundle (CeramOptec GmbH). The ultrasound detection probe was composed of an acoustic couplant and a cylindrically focused 256-element detector array (center frequency, 4 MHz; send/receive bandwidth, 60%; resolution, ~200 µm) with 135° coverage to provide 2D cross-sectional images with a field of view of 25 × 25 mm [2] and a reconstructed pixel size of 62.5 µm. Three versions of the probe involving different couplants were tested during the study: 1) water; 2) first generation solid couplant, custom-designed by iThera to improve usability; 3) second generation solid couplant with improved long-term stability. The technology was being developed during the study, hence the changes in prototype system and probe couplants.

Both prototype systems also included low frequency (4 MHz) tomographic ultrasound using the curvilinear ultrasound probe as described previously [27], which enabled accurate lesion location within OA-US images. Diagnostic US examinations were also performed using an Accuson S2000 US scanner (18 L6 HD transducer, Siemens Medical Solutions). Mammography was performed using GE mammography systems. The US and mammography were part of the patients’ standard of care performed by board certified breast radiologists and mammographers within our breast service.

All patients were given routine standard of care regardless of the outcome of OA-US. Simple cysts were differentiated from solid lesions on diagnostic B-mode US. They were aspirated for symptom relief or managed conservatively with reassurance. For solid lesions in patients over the age of 30, the gold standard was histopathology i.e. 14 G core biopsy. Solid lesions in women under the age of 30, which fulfilled the Stavros criteria [8], were classified as benign.

OA-US images were reconstructed using a standard back-projection algorithm [28] after band-pass filtering and deconvolution with the electrical impulse response of the transducer. Images were analysed using cLabs 2.59 software (iThera Medical GmbH, Germany). Fluence correction was not used for data acquired with the MSOT-EIP (feature definition set) but it was used for the feature validation data acquired with the MSOT Acuity Echo prototype according to previously published methods [27].

### 2.3 Development of the feature set

The integrated light source and detector array probe had a curvilinear configuration and was positioned on the skin of the breast similar to the position of a routine US probe. Hence, the morphological detail was seen in the upper part of the image at the anterior and lateral margins of a breast lesion. We chose a single wavelength of 800 nm (isosbestic point of oxy- and deoxy-haemoglobin) to obtain an image reflective of the morphology of blood vessels surrounding a solid breast lesion to complement the BI-RADS US lexicon [29] for lesion characterisation.

Images of solid benign and malignant lesions from the first part of the study were reviewed by authors (OA, SM, ND, SEB and FJG). The OA and US images were considered separately, then in combination, as the hybrid image of OA and B-mode US can be used to understand the anatomical relationship between the OA data and the lesion position. The ‘feature development team’, which included two experienced breast radiologists, looked for patterns in keeping with known appearances of blood vessels in healthy breast tissue, benign disease and malignancy (hallmarks of cancer). The patterns observed were interpreted in the context of the probe configuration and histopathology of the lesion. Descriptive terminology from a radiologist’s vocabulary was used to describe the patterns seen with OA-US. The patterns unique to benign and malignant lesions were considered a sign of malignancy or benignity.

### 2.4 Validation of the feature set

A different senior breast radiologist, with over 25 years of breast experience and a different junior radiologist, with 5 years of experience in breast imaging, acted as “readers”. Neither of these radiologists were involved in creating the feature set or had prior experience in OA imaging; they were also blinded to the histopathology / clinical findings. They were given a tutorial on OA imaging and shown examples from cases used to generate the feature set. The readers were first asked to use the features set described in the methods section to classify lesions as BI-RADS 2 or BI-RADS 4 or 5. They then independently and blindly scored the US and mammography using the BI-RADS lexicon and included the assessment of breast density. Both readers blindly and independently read the OA-US studies first, a week later they scored the US, and two weeks later they scored the mammograms. OA-US images were presented in the order they were acquired. Mammograms and US images were presented in a random order to minimise any memory biasing of the result.

### 2.5 Statistical analysis

To calculate the sample size for the validation/reader study, the A'Hern [30] method was used. To test a correct rate < 80% vs > 95%, with a significance level of 5% and power of 90%, a sample size of 44 lesions was required. The sensitivity and specificity of mammography, US and OA-US were calculated independently for the senior and junior radiologists. True positives were defined as a BI-RADS 4a-c and 5 confirmed as malignant on biopsy. A true negative was defined as BI-RADS 2: benign biopsy or Stavros criteria of benignity [8]. A false positive was defined as BI-RADS 3 – 5 with benign biopsy. A false negative was defined as a BI-RADS 1–3 with positive biopsy. BI-RADS 3 lesions are ‘Probably benign’, which means there is a small (<2%) chance of malignancy, making them challenging to assess. In our study, there were 3 BI-RADS 3 lesions identified by mammography, 7 by ultrasound and none by OA-US. BI-RADS 3 lesions were included into the false positive grouping but not the true positive grouping because of their small probability of malignancy.

## 3 Results

**Fig. 1:**
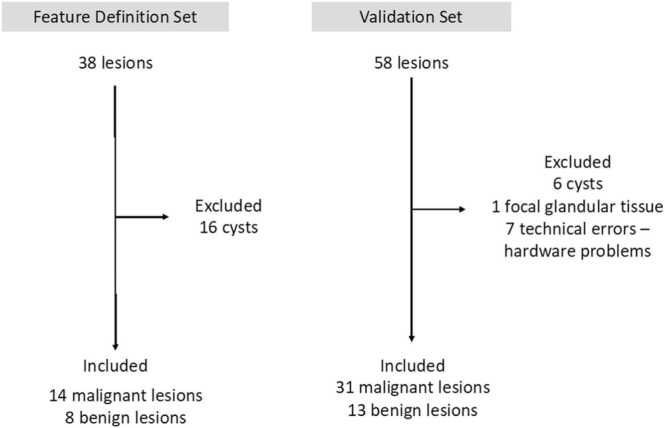
Summary of lesions included in the feature definition and validation sets. Cysts were excluded from both sets. In the validation set, there was 1 case with focal glandular tissue that was excluded and in addition there were errors in data acquisition on 7 occasions that rendered the data unusable.

A total of 94 patients with 96 lesions were recruited for the study. All solid or partially solid masses imaged were included in the study (Fig. 1). The first 38 lesions scanned were used for the development of the feature set, within which 14 malignant and 8 benign solid lesions were represented; the rest were simple cysts. The subsequent 44 lesions were used for the validation set, 31 malignant and 13 benign. The age range, menopausal status and histopathology of lesions are summarised in Table 1. The lesion size ranged from 3 mm to 70 mm on histopathology.

**Table 1:** Summary of the patient characteristics for the lesions included in the feature definition and validation sets. Abbreviations: OCP, oral contraceptive; HRT, hormone replacement therapy; NST, invasive breast cancer, no special type; ILC, infiltrating lobular carcinoma; DCIS, ductal carcinoma in situ; FA, fibroadenoma.

		**Feature Definition Set**	**Validation Set**
**Age of patients**		28 – 88 (mean = 58.5)	24 – 86 (mean = 59.7)
**Menopausal status of patients** Pre-menopausal Post-menopausal On OCP, Coil, HRT Peri-menopausal Unknown		7/22 (31.8%) 13/22 (59.1%) 2/22 (9.1%) 0/22 (0%) 0/22 (0%)	12/42 (28.6%) 24/42 (57.1%) 3/42 (7.1%) 2/42 (4.8%) 1/42 (2.4%)
**Histopathology** Malignant lesions NST Grade 1 NST Grade 2 NST Grade 3 ILC Grade 2 ILC Grade 3 DCIS high grade Other (papillary, mucinous) Benign lesions FA Fibrocystic change Scar tissue / fat necrosis Sclerosing adenosis Complex cyst Lipoma		0/14 (0%) 7/14 (50%) 4/14 (28.6%) 0/14 (0%) 1/14 (7.1%) 2/14 (14.3%) 0/14 (0%) 5/8 (62.5%) 1/8 (12.5%) 1/8 (12.5%) 1/8 (12.5%) 0/8 (0%) 0/8 (0%)	2/31 (6.5%) 15/31 (48.4%) 8/31 (25.8%) 2/31 (6.5%) 2/31 (6.5%) 0/31 (0%) 2/31 (6.5%) 10/13 (77.0%) 0/13 (0%) 1/13 (7.7%) 0/13 (0%) 1/13 (7.7%) 1/13 (7.7%)

### 3.1 Development of the feature set

**Fig. 2:**
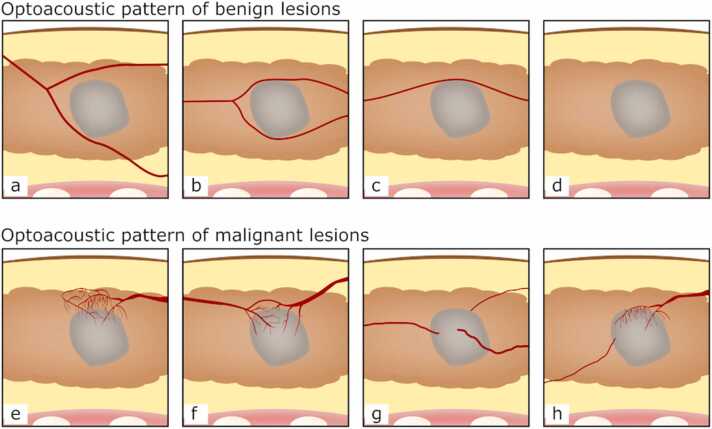
Schematic representation of OA patterns observed in benign and malignant lesions. Benign: a) splayed vessels sign, b) and c) vessels draped over the lesion sign, d) absent vessel sign. Malignant: e) irregular cap sign, f) claw sign, g) irregular feeding vessel sign and h) irregular feeding vessel and an irregular cap sign.

**Fig. 3:**
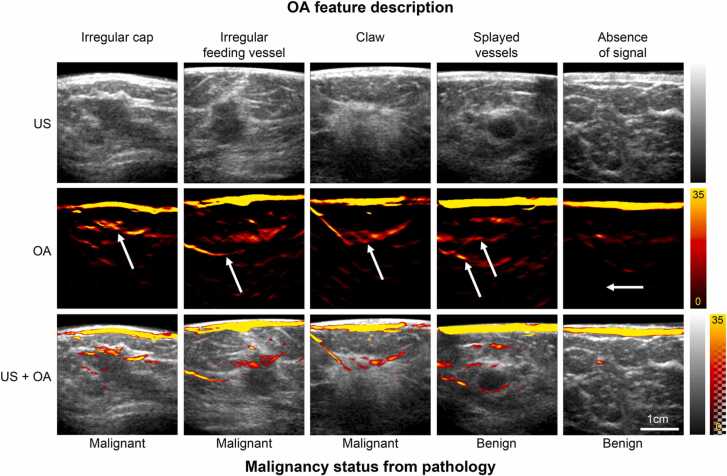
OA-US images illustrating the patterns used to define the feature set for benign and malignant lesions. OA features are specified above and lesion histopathology below the image panels. Arrows indicate the specific OA feature relative to the lesion, visible in the US image and OA-US.

**Fig. 4:**
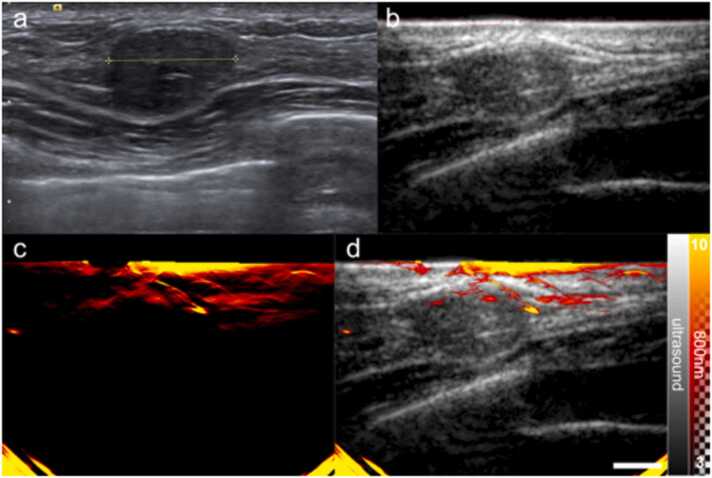
Exemplar fibroadenoma (benign). (a) Standard-of-care ultrasound reveals an oval hypoechoic mass, without lobulations; the mass is greater in width than height. (b) The US image from the OA-US scan also reveals an oval hypoechoic mass (c) The OA image from the OA-US scan at 800 nm reveals blood vessels that appear to be draped over the lesion. (d) Overlaid OA-US image highlights the relationship between the visualised blood vessels and the benign mass. Scale bar 1 cm.

**Fig. 5:**
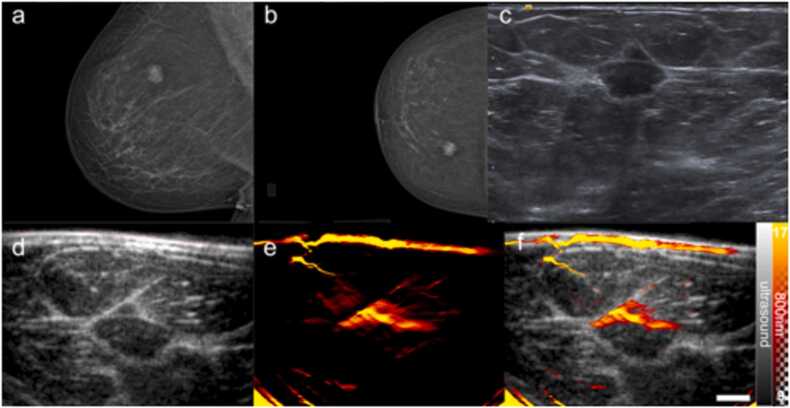
Exemplar grade 2 invasive ductal carcinoma (ER+ PR+ Her2-). Mammography [MLO (a) and CC (b) views] reveal a lobulated mass in the upper inner quadrant of the left breast. The outline has a few spicules. (c) Standard-of-care ultrasound reveals a hypoechoic irregular mass. (d) The US image from the OA-US scan also reveals an irregular hypoechoic mass, albeit at lower resolution than the clinical US imaging. (e) The OA image from the OA-US scan at 800 nm shows an irregular cap of signal at the anterior margin of the cancer (e) Overlaid OA-US image highlights the relationship between the visualised neoangiogenesis from OA and tumour mass position on US. Intense OA signals at the breast surface arise due to absorption in the skin by haemoglobin in the skin microvasculature as well as melanin pigmentation. Scale bar 1 cm.

The OA-US observations of the blood vessels surrounding benign and malignant lesions contained within the feature set correlated with the expected biology. Benign lesions demonstrated no vascularity, or vessels that splayed / draped over the lesion without penetrating into it (Fig. 2a-d). Malignant lesions included irregular feeding vessels that penetrated into the lesion and/ or a disorganised irregular pattern of vessels around the malignant lesions (Fig. 2e-h), as would be expected from a lesion that has stimulated neoangiogenesis. The internal appearances of the lesions, though considered, were not included in the final feature set as they were not helpful in the differentiation of benign and malignant lesions. Exemplar OA-US images from all signs are illustrated in Fig. 3; a specific example of a fibroadenoma is shown in Fig. 4, while an exemplar from a grade 2 invasive ductal carcinoma is shown in Fig. 5.

Three features of malignancy were selected to upgrade any solid lesion to a BI-RADS 5 lesion: irregular cap, irregular feeding vessel and claw sign. Two features of benignity were created to downgrade a lesion to a BI-RADS 2 lesion: no vessels present and vessels splayed/draped over the lesion vessel. When the developed feature set was applied to the 22 cases used to create it, 13/14 malignant and 7/8 benign lesions were correctly diagnosed. The false positive was a case of sclerosing adenosis and the false negative case was a lobular invasive carcinoma.

### 3.2 Performance of the identified features in the validation set

Having established the feature set, two readers were trained and exposed to the validation set. The junior reader (5 years experience) reported sensitivities of 90.3%, 96.8%, 96.8% for mammography, US, and OA-US, respectively, with associated specificities of 75.0%, 53.8%, and 84.6%. The senior reader (25 years experience) reported related sensitivities of 90.3%, 96.8%, 96.8% and specificities of 75%, 46.1%, and 84.6%. The results are summarised in Table 2. Mammography yielded 3 false negatives and 2 false positives for each reader. US had fewer false negatives, one per reader, but more false positives than mammography. The senior reader had seven false positives and the junior reader had six false positives. OA-US resulted in 1 false negative and 2 false positives for both readers.

**Table 2:** Summary of sensitivity and specificity values for the standard-of-care mammography and US, compared to OA-US.

**Imaging Modality**		**Junior Reader**	**Senior Reader**
Mammography	Sensitivity (%)	90.3	90.3
	Specificity (%)	75.0	75.0
US	Sensitivity (%)	96.8	96.8
	Specificity (%)	53.8	46.1
OA-US	Sensitivity (%)	96.8	96.8
	Specificity (%)	84.6	84.6

**Fig. 6:**
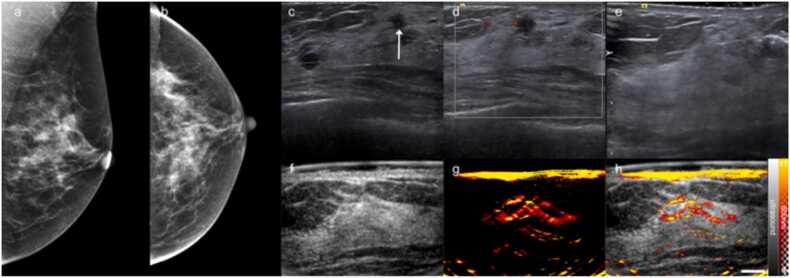
A false negative from the standard-of-care imaging that was successfully upgraded by OA-US. Mammography [MLO (a) and CC (b) views] do not indicate breast cancer (c) Standard-of-care ultrasound reveals a few scattered cysts and a small subcentimetre irregular hypoechoic mass in a retroareolar position. (d) Ultrasound imaging with Doppler demonstrates two small vessels. (e) Alternate view from ultrasound imaging without Doppler (f) The US image from the OA-US scan at the same position does not clearly reveal the irregular hypoechoic mass seen on US compared to the background breast parenchyma. (g) The OA image from the OA-US scan at 800 nm reveals a lattice of high signal similar to the irregular cap sign seen in malignancy, which would upgrade the lesion to BI-RADS 5. (h) Overlaid OA-US image. Scale bar 1 cm.

Importantly, the false negatives recorded by mammography and US were all correctly identified as positive by OA. An exemplar of such a case from the validation set is shown in Fig. 6, where breast cancer was not apparent on mammography, but an irregular hypoechoic mass could be observed using ultrasound. In the OA+US image, an irregular cap of signal was visible, hence readers confidently upgraded the classification to BI-RADS 5. Core biopsy revealed a grade 2 lobular invasive carcinoma (ER+ PR+ Her2 -). Lobular carcinoma is an infiltrative tumour which does not always form a mass. It is often difficult to detect on conventional imaging such as mammography and US.

In 3 of the 4 cases for which a false negative was recorded by either US or mammography, breast density scores of BI-RADS C were recorded, suggesting that a masking effect could have contributed to the inaccuracy of these results. OA correctly scored all four of these cases, suggesting that OA might not be susceptible to masking effects related to dense breast tissue.

**Fig. 7:**
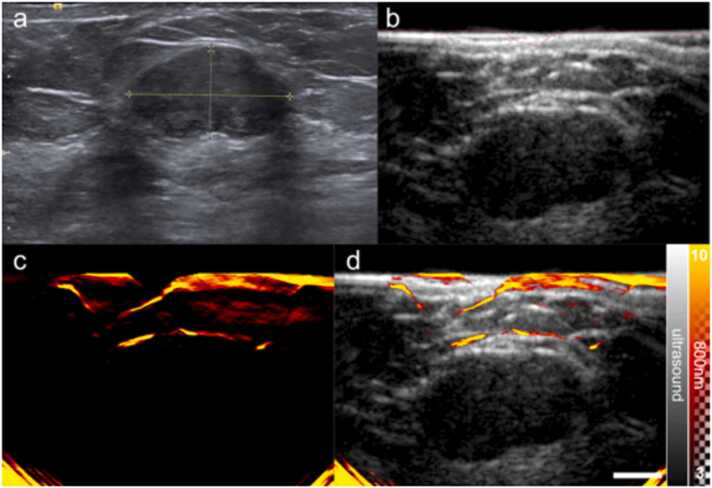
A BI-RADS 3 lesion successfully downgraded by OA-US. (a) Standard-of-care ultrasound reveals an oval hypoechoic mass, with few lobulations seen, hence on B mode US alone the lesion was classified as BI-RADS 3. (b) US image from the OA-US scan at the same position reveals an oval hypoechoic mass (c) The OA image from the OA-US scan at 800 nm reveals blood vessels, which appear draped over the lesion in the overlaid OA-US image (d) and would downgrade the image to BI-RADS 2. Scale bar 1 cm.

In addition to correctly upstaging a lesion that was a false negative from the standard-of-care, OA-US was also able to correctly downgrade a lesion in the validation set from BI-RADS 3 (probably benign) to BI-RADS 2 (benign), as illustrated in Fig. 7. The lesion was diagnosed as a fibroadenoma.

**Fig. 8:**
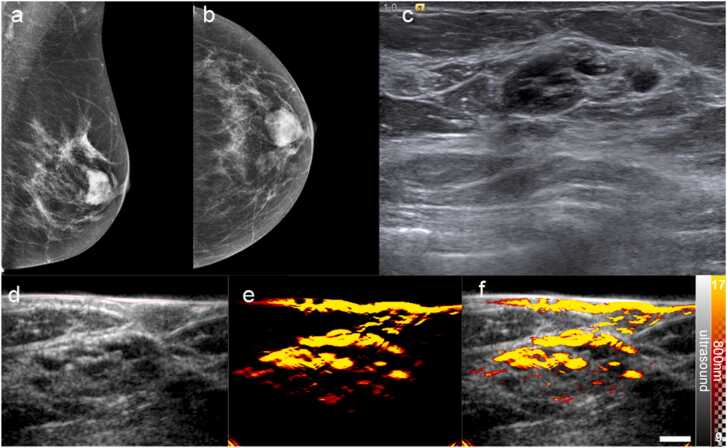
A false positive on OA-US. Mammography [MLO (a) and CC (b) views] reveals an oval mass in a retroareolar position. (c) Standard-of-care ultrasound reveals a complex cyst with multiple septations and a thick wall. (d) The US image from the OA-US scan at the same position is similar to the B Mode US. (d) The OA image from the OA-US scan at 800 nm reveals a pattern similar to that of disorganised vessels, which was interpreted as an irregular cap sign of malignancy, and the lesion was upgraded to malignant. (e) Overlaid OA-US image highlights the relationship between the area of high signal and the complex cyst. Scale bar 1 cm.

Although OA-US provided a correct scoring for false negatives from the standard-of-care imaging, there were also 2 false positive cases. An exemplar from the case of a complex cyst is shown in Fig. 8, where OA-US incorrectly led to upgrading of the lesion to malignant. False positives have previously been reported in complex cysts because the presence of water can lead to distortions that give rise to a range of artefactual signals that mimic the appearance of tumours [31].

## 4 Discussion

Optoacoustic imaging has shown promise for application in breast imaging, but current feature definitions used to delineate benign and malignant lesions are complex and hence may be challenging to apply in practice. Our study demonstrated that a simple feature set derived using data acquired with an OA-US system could improve the identification of benign and malignant breast disease when compared to mammography and standard-of-care ultrasound. Our results also suggest the potential to improve the detection of breast cancer in the clinic in the challenging subpopulation of patients with dense breasts, a preliminary observation that should be examined in more detail in future studies. Importantly, these findings were obtained using only a single excitation wavelength, which affords the possibility for future system simplification and cost reduction.

The OA-US sensitivity in our study was similar to previous findings in clinical breast OA-US studies (96.8% in this study vs 98% and 95.5% in prior studies) [13], [14], however, our specificity was much higher (84.6% vs 43% and 41.1%) [13], [14]. The reasons for our elevated specificity could be multifactorial. For example, our inclusion criteria included lesions that ranged from BI-RADS 1–5 to reflect clinical practice, rather than 4a alone as used in the prior studies. In addition, the scoring system used in our study is much simpler than that used in the referenced prior studies, where six features were divided into five categories, yielding 30 used signs [13], [14]. In contrast, our study provided readers with 3 signs for upgrading and 2 signs for downgrading a lesion, considerably simplifying the clinical diagnostic process. Our feature sets for benign and malignant disease were created with a focus on simplicity of application using only single wavelength data.

In addition, there were significant technical differences between the OA-US imaging platforms used. The curvilinear US detectors used here enable limited view tomography, typically affording improved spatial resolution for a given frequency and more accurate depiction of continuous features such as vascular branching, which may have aided the development and use of the feature set in the present study. Furthermore, the use of a single wavelength further simplified the processing and visualisation compared to prior studies. It also removed the need for linear spectral unmixing into different absorbers, which can be problematic at depth in tissue due to spectral colouring [32]. We also focused on data acquisition at 800 nm, which minimises absorption by water and lipids [16]. The result was complete agreement between readers with respect to benign or malignant status using OA-US, regardless of the experience of the reader. Another important advantage of this approach, in addition to the simplicity of the feature sets, is that readers can be trained quickly (i.e. 20 min in this study) in recognising the patterns of benignity or malignancy.

There were several limitations to our study. Firstly, although our sample size was sufficient based on our power calculation, it was still relatively small so under-sampling could have created a bias in the results. As we scanned consecutive patients who consented into the study, there was a bias in the ratio of benign and malignant lesions. Furthermore, we conducted a reader study, which eliminated the operator learning curve of acquiring OA-US images. OA-US is a handheld technique similar to US, which requires practical experience to optimise the standard operating procedure and obtain high quality image data. Future studies should also consider operator dependence, as well as the practicalities of installing OA-US systems for safe operation, data acquisition and appropriate interpretation of images.

Our study highlights some more general limitations of OA in the assessment of breast lesions, which have also been noted in the prior literature. If understood, future studies evaluating the positive contribution of OA to clinical pathways could be facilitated. False-positive results were related to complex masses with both solid and cystic components. These are often classified as indeterminate even on mammography and US. Cysts can have variable appearances in OA due to the absence of vascular structures. Cystic lesions should not be down-graded or upgraded by OA alone, however, as simple cysts can be differentiated from solid masses by B mode US and the solid components of complex cysts can be targeted during US guided intervention [31], a lack of OA signal could be used to support US assessment. The cancer missed with OA was an invasive lobular cancer (ILC); no optoacoustic signal was observed. ILC is an infiltrative tumour known to be challenging to detect on most clinical imaging modalities and we also presented here a case where OA-US was able to detect an ILC that was not identified with mammography or US. The inability of OA to identify this other case of ILC may be because the tumour did not form a mass or recruit vessels in the same manner as other histopathological subtypes.

In conclusion, we have developed a simple feature set that was able to improve the detection of breast cancer using OA-US when applied by two independent readers. The simple feature set and single wavelength acquisition would lend itself to potentially creating a low-cost device that could be applied in low resource settings and hence highlights the potential of OA-US across a range of healthcare settings for breast cancer management. In particular, readers were rapidly trained to recognise the different patterns from the feature set and both junior and senior readers performed equally. The diagnostic relevance of our proposed feature set in clinical practice needs to be validated in larger multi-centre multi-reader trials.

## Declaration of Competing Interest

The authors declare the following financial interests/personal relationships which may be considered as potential competing interests, Sarah Bohndiek reports a relationship with EPFL Center for Biomedical Imaging that includes: speaking and lecture fees. Sarah Bohndiek reports a relationship with PreXion Inc that includes: funding grants. Sarah Bohndiek reports a relationship with iThera Medical GmbH that includes: non-financial support. Stefan Morscher, Nina Dalhaus, Steven Ford and Neal Burton report a relationship with iThera Medical GmbH that includes: employment. Fiona Gilbert reports a relationship with Google and Kheiron that includes: consultancy. Fiona Gilbert reports a relationship with PreXion Inc, GE Healthcare, Bayer and Hologic that includes: funding grants. Fiona Gilbert reports a relationship with Lunit, Screenpoint, Volpara, iCAD, Therapixel and Vara that includes: research collaboration.

## Data Availability

Data associated with this study can be made available upon request to the corresponding authors SEB or FJG.
